# Advancements in the production of value-added products via methane biotransformation by methanotrophs: Current status and future perspectives

**DOI:** 10.71150/jm.2412024

**Published:** 2025-03-28

**Authors:** Ok Kyung Lee, Jong Seok Lee, Yoonyong Yang, Moonsuk Hur, Kyung Jin Lee, Eun Yeol Lee

**Affiliations:** 1 Department of Chemical Engineering (BK21 FOUR Integrated Engineering), Kyung Hee University, Gyeonggi-do 446-701, Republic of Korea; 2 National Institute of Biological Resources(NIBR), Incheon 22689, Republic of Korea

**Keywords:** methanotrophs, methane, bioconversion, value-added products

## Abstract

Methane gas is recognized as a promising carbon substrate for the biosynthesis of value-added products due to its abundance and low price. Methanotrophs utilized methane as their sole source of carbon and energy, thus they can serve as efficient biocatalysts for methane bioconversion. Methanotrophs-catalyzed microbial bioconversion offer numerous advantages, compared to chemical processes. Current indirect chemical conversions of methane suffer from their energy-intensive processes and high capital expenditure. Methanotrophs can be cell factories capable of synthesizing various value-added products from methane such as methanol, organic acids, ectoine, polyhydroxyalkanoates, etc. However, the large-scale commercial implementation using methanotrophs remains a formidable challenge, primarily due to limitations in gas-liquid mass transfer and low metabolic capacity. This review explores recent advancements in methanotroph research, providing insights into their potential for enabling methane bioconversion.

## Introduction

Methane, the major component of biogas and natural gas, serves as a valuable feedstock for producing chemicals and fuels due to its high energy density and simple molecular structure (Clomburg et al., 2017; Ghasemi Ghodrat et al., 2018). However, methane is also a powerful greenhouse gas with a global warming potential (GWP) that is 27 to 30 times greater than that of carbon dioxide over a 100 year period (GWP of carbon dioxide is defined as 1) (EPA, 2024). In 2020, atmospheric methane concentrations attained 1892.3 ppb, indicating a 0.78% increase from the previous year and constituting the most significant yearly rise since record-keeping commenced in 1983 (NOAA, 2021). As atmospheric methane levels continue to rise due to anthropogenic activities such as agriculture, fossil fuel extraction, and waste management, innovative approaches are urgently needed to reduce methane emissions while deriving economic value (Ghasemi Ghodrat et al., 2018; Lee et al., 2016).

While conventional approaches to methane conversion primarily rely on chemical processes, such methods often require high temperatures, pressures, and expensive catalysts, resulting in limited product diversity and significant energy costs (Fei et al., 2014). In contrast, biological methane conversion using methanotrophs—a specialized group of microorganisms capable of oxidizing methane as their sole carbon and energy source—has gained traction as a sustainable and versatile alternative (Fei et al., 2014; Lee et al., 2016; Strong et al., 2016; Tan et al., 2024). Methanotrophs offer unique advantages for methane valorization, operating under mild conditions to produce a wide range of value-added products such as single-cell protein (SCP), biodegradable polymers like polyhydroxyalkanoates (PHAs), organic acids, biofuels, and other biomaterials (Cantera et al., 2018a; Gęsicka et al., 2021; Kang et al., 2024a; Lee et al., 2016; Liu et al., 2020; Pieja et al., 2017). Additionally, methanotrophs can utilize methane from both pure sources and gas mixtures, such as biogas, which contains impurities like carbon dioxide, hydrogen sulfide, alkane, alkene, etc (Comesaña-Gándara et al., 2022; Henard et al., 2018; Hyun et al., 2024; Patel et al., 2020; Rodríguez et al., 2020a, 2020b).

Over the past decade, significant strides have been made in understanding methanotroph biology and harnessing their metabolic potential. Advances in genome sequencing and transcriptomic of methanotrophs have provided valuable insights into their metabolic pathways (Naizabekov et al., 2023; Nguyen et al., 2018, 2019a; Oshkin et al., 2021; Sugden et al., 2021; Vorobev et al., 2014). Concurrently, advancements in genetic engineering tools have allowed researchers to modify methanotrophs, leading to the creation of strains to produce various value-added products derived from methane (Ishikawa et al., 2018; Jeong et al., 2023; Kang et al., 2024b; Lee et al., 2021; Nguyen et al., 2019b; Puri et al., 2015; Rumah et al., 2023; Tapscott et al., 2019; Yan et al., 2016).

This review overviews methanotrophs and their metabolic pathways and offers a detailed description of recent progress in methanotroph-based production of value-added compounds, focusing on advances in metabolic engineering. By highlighting key developments over the past decade, we seek to identify both the opportunities and challenges in scaling up methane bioconversion technologies for industrial applications.

## Classification of methanotrophs

Methanotrophs are bacteria that derive energy by oxidizing C1 substrates, including methane and methanol, in both aerobic and anaerobic conditions. Despite the initial identification of methanotrophs in 1906, a significant breakthrough occurred in 1970 when Whittenbury et al. effectively isolated and characterized over 100 gram-negative methane-oxidizing bacteria (MOB) strains (Hanson & Hanson, 1996; Wendlandt et al., 2005).

These MOBs are prevalent in diverse environments, particularly in ecosystems characterized by methane emissions, including wastewater sludge, freshwater and marine debris, wetland ecosystems, coal and oil deposits, wastewater treatment plants, and biogas facilities. Furthermore, they can be extracted from severe settings marked by temperature, pH, or salinity fluctuations (Knief, 2015; Strong et al., 2015).

**Fig. 1.**
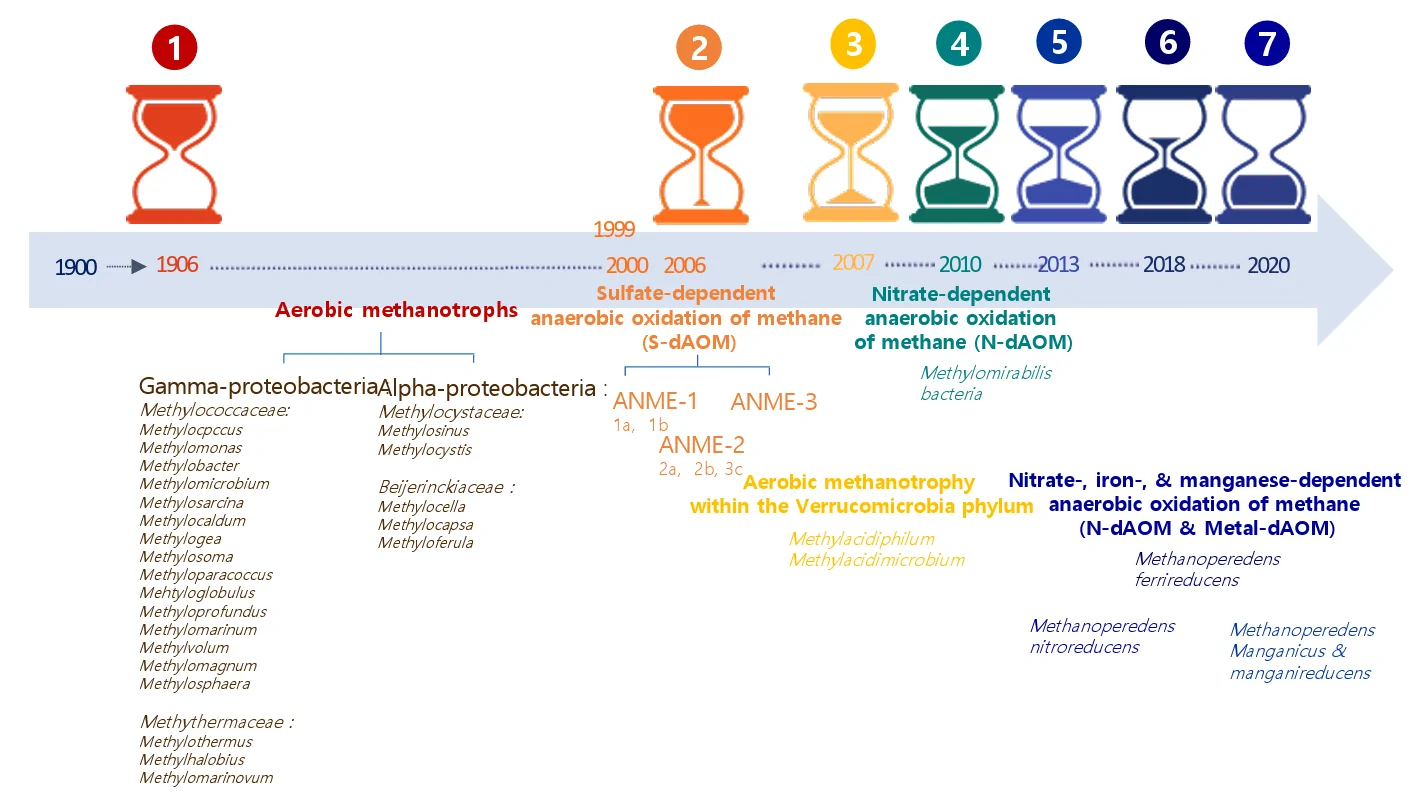
120 years of methanotrophs: a timeline of key milestones and phylogenetic discoveries.

Aerobic methanotrophs were thought to be the only biological mechanism for methane oxidation for nearly a century. However, over the past 20 years, discovering microorganisms that can oxidize methane under anaerobic conditions using alternative electron acceptors such as sulfate, nitrite, nitrate, iron, and manganese has broadened the scope of known methanotrophs (Guerrero-Cruz et al., 2021). Figure 1 provides a temporal summary of aerobic and anaerobic methanotrophs. It illustrates a 120-year chronology of methanotrophs, with each section of the arrows representing a generation. This emphasizes the evolutionary classification of various methanotroph groupings. Methanotrophs are categorized into seven principal milestones, meaning the most significant microbiological discoveries. The red-marked group in Fig. 1 identifies aerobic methanotrophs in 1906, with all recognized genera enumerated according to their evolutionary classification (Dedysh & Knief, 2018). The orange-highlighted group signifies the identification of sulfate-dependent anaerobic oxidation of methane (S-dAOM), a mechanism executed by three distinct groups of anaerobic methanotrophic archaea (ANME) within the *Euryarchaeota*. The yellow-highlighted organisms are aerobic methanotrophs classified within the phylum *Verrucomicrobia*. The regions marked in green indicate nitrate-dependent anaerobic methane oxidation (N-dAOM) performed by *Methylomirabilis* bacteria. The areas highlighted in dark-blue denote nitrate, iron, and manganese-dependent anaerobic oxidation (N-dAOM and Metal-dAOM) conducted by various cultivated species of the family *Methanoperedenaceae*.

Methanotrophic bacteria are classified into three phyla: Proteobacteria, *Verrucomicrobia*, and the recently recognized candidate phylum NC10 (Kalyuzhnaya et al., 2019). Within the proteobacteria, aerobic methanotrophs are split into two main groups: gamma-proteobacteria and alpha-proteobacteria. Gamma-proteobacteria encompass various aerobic methanotrophic taxa, including *Methylomonas*, *Methylococcus*, *Methylomicrobium*, *Methylosarcina*, *Methylocaldum*, and *Methylobacter*. Alpha-proteobacteria consists of four genera: *Methylosinus*, *Methylocystis*, *Methylocella*, and *Methylocapsa* (Fig. 1) (Guerrero-Cruz et al., 2021). Despite their diversity, methanotrophs possess a unified metabolic route that oxidizes methane to carbon dioxide via formaldehyde.

## Methane Metabolism of Methanotrophs

Methane metabolism in methanotrophs begins with the conversion of methane to methanol, a reaction catalyzed by methane monooxygenase. The presence and activity of this reaction are defining characteristics of methanotrophs (Hanson & Hanson, 1996).

Methanotrophs utilize their methane monooxygenase complexes to break the strong C-H bond in methane, facilitating its oxidation at normal temperature and pressure in the presence of oxygen (Hwang et al., 2014; Lee et al., 2016). Methanotrophs can express two varieties of methane monooxygenases: the membrane-associated particulate methane monooxygenase (pMMO) and the cytoplasmic soluble methane monooxygenase (sMMO) (Hanson & Hanson, 1996; Hwang et al., 2014; Lee et al., 2016). Methanotrophs can express either one or both methane monooxygenases, with pMMO being the more commonly found form. While sMMO exhibits broad substrate specificity, its expression is suppressed under high copper concentrations, and only a few methanotrophs carry this enzyme. In species that possess both pMMO and sMMO, copper concentration is a critical determinant of both enzymes' gene expression and activity (Hanson & Hanson, 1996).

**Fig. 2.**
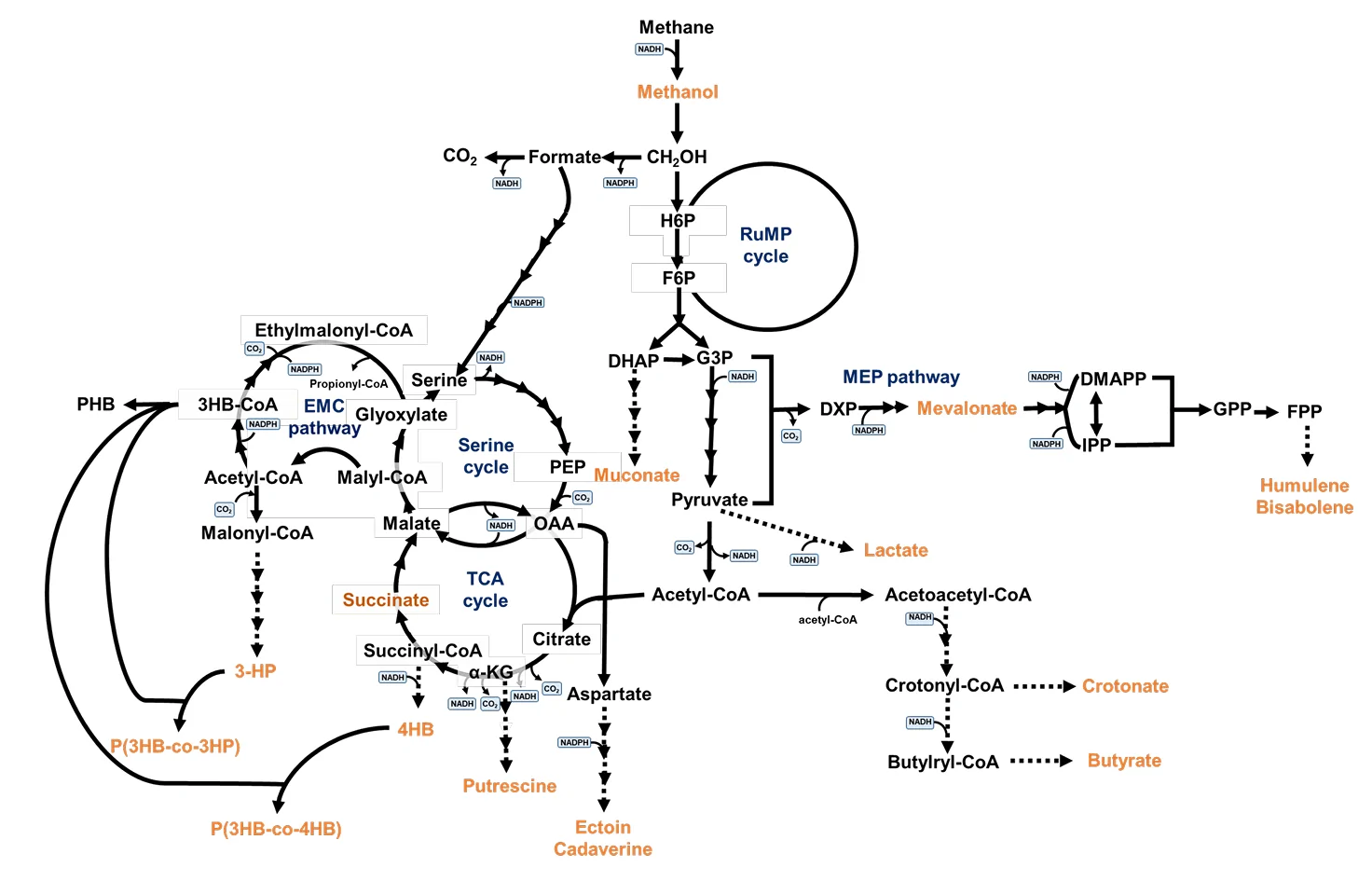
Central carbon metabolism of methanotrophs and metabolic pathways for target products derived from methane.

After methane is first converted to methanol, methanol is oxidized to produce formaldehyde, which is further converted into formate and CO_2_ (Fig. 2) (Hanson & Hanson, 1996). The produced formaldehyde can either be: i) further oxidized to CO_2_ or ii) assimilated via the ribulose monophosphate (RuMP) cycle, while formate is assimilated through the serine cycle. In the process of converting formaldehyde to CO_2_, formaldehyde is oxidized to formate by formaldehyde dehydrogenase (FADH), after which formate is further converted to CO_2_ by formate dehydrogenase (FDH). The RuMP and serine cycles generate pyruvate, which is metabolized to acetyl-CoA, a key intermediate for cellular metabolism. In the RuMP cycle, formaldehyde is converted into fructose-6-phosphate through catalytic reactions mediated by hexulose phosphate synthase and phosphohexose isomerase (Fig. 2).

Methanotrophs that assimilate C1 substrates through the serine cycle initially incorporate formate into the serine cycle via the H_4_F pathway, where C1 substrates are transformed into C3 and C4 molecules, subsequently converted to acetyl-CoA, which enters the TCA cycle (Hanson & Hanson, 1996; Lee et al., 2016). One of the key distinguishing features between methanotrophs is their C1 carbon metabolism mechanism, either via the RuMP or the serine cycle, which forms the basis for classification (Fei et al., 2014; Hanson & Hanson, 1996; Lee et al., 2016). Methanotrophs employing the RuMP cycle for carbon fixation are classified as gamma-proteobacteria (type I), whereas alpha-proteobacteria (type II methanotroph) utilize the serine cycle. Various methanotrophs exhibit differences in the positioning of their intracytoplasmic membranes, nitrogen-fixing capabilities, and the composition of their predominant phospholipid fatty acids.

The growth of methanotrophs is greatly affected by environmental conditions, including temperature, pH, methane and oxygen levels, metal ions, and nutrients. Among these, temperature and pH are the most influential factors. Most methanotrophs grow at temperatures between 20°C and 35°C, though certain strains can grow in extreme temperatures. For instance, *Methylothermus* sp. and *Methylacidiphilum* species can grow at elevated temperatures of 55°C to 60°C (AlSayed et al., 2018). Regarding pH, most methanotrophs (over 90%) prefer a range of 5.5 to 8.0 for optimal growth. However, methanotrophs from the phylum *Verrucomicrobia* are specialized for acidic environments, showing best growth at pH levels of 1.5 to 3.5. Acidophilic strains such as *Methylacidiphilum fumariolicum* and *Methylacidimicrobium* sp. are even capable of growing at extremely low pH levels of 0.5 to 0.8 (AlSayed et al., 2018). Another key factor influencing methanotrophs' growth is methane and oxygen concentration. Type I methanotrophs efficiently grow and oxidize methane under high concentrations of oxygen and methane, while type II methanotrophs can adapt to low concentrations of oxygen and methane but exhibit lower methane conversion efficiency (Lee et al., 2016). Among metal ions, copper is well known as a major factor affecting methanotroph growth and methane oxidation. Copper is crucial for regulating the expression of two forms of MMO (DiSpirito et al., 2016). Under copper-limited conditions, sMMO is expressed, whereas pMMO requires copper for its expression. Most type I methanotrophs contain pMMO, and studies have shown that adding 2–10 μM of copper can enhance methanotrophs’ growth and methane oxidation rate. For nitrogen sources, methanotrophs commonly utilize nitrate and ammonium (Bowman et al., 2016; Lopez et al., 2013). MMO can convert ammonium into hydroxylamine, which may partially inhibit methanotroph growth. On the other hand, nitrate promotes the initial growth rate of both type I and type II methanotrophs more effectively than ammonium (Karthikeyan et al., 2016). The specific growth rates of methanotrophs vary depending on their type under optimal cultivation conditions. For type I methanotrophs, the maximum growth rate has been reported as 0.3 h^⁻1^, whereas type II methanotrophs exhibit a lower maximum growth rate of approximately 0.15-0.18 h^⁻1^ (Dedysh et al., 2007).

## Potential of Methanotrophs as a Platform for Methane Bioconversion

Methanotrophs have historically been regarded as a valuable platform for methane bioconversion, exhibiting significant biotechnological promise. They are currently being employed industrially for the commercial production of several goods, including PHA pellets, animal feed protein (Calysta, 2014; Circumfauna, 2024; Pieja et al., 2016; Plastics engineering, 2024) The increasing interest in methanotroph applications is evidenced by the rising number of research articles and patents dedicated to converting methane into high-value products. Type I and type II methanotrophs have been investigated as platforms for methane bioconversion. Over the past decade, much research has been conducted on utilizing these organisms to generate diverse high-value chemicals, particularly in the latter half of the decade, and on enhancing the productivity of these valuable chemicals through methanotrophs.

Research utilizing wild-type methanotrophs has made significant progress in optimizing the synthesis efficiency of methanol, biopolymers, ectoine, and animal feed protein from methane. Furthermore, the development of genetic tools applicable to methanotrophs has not only improved the production of naturally occurring compounds but also enabled the metabolic engineering of these organisms for the biosynthesis of non-natural high-value compounds such as organic acids, secondary metabolites, and other compounds. In the following section, we aim to provide a comprehensive summary of the technological advancements in genetic tools for methanotrophs over the past decade and the resulting achievements in the biosynthesis of high-value compounds.

## Advances in Genetic Tools for Metabolic Engineering of Methanotrophs

To support the metabolic engineering of methanotrophs, various genetic tools have been developed. These tools include plasmids for heterologous gene expression, plasmids for the deletion and insertion of specific genes in the chromosome, and gene transfer methods to introduce these plasmids into methanotrophs. The advancement of NGS technology has made whole-genome analysis of type I and type II methanotrophs more accessible, facilitating the rapid development of genetic tools for these organisms. Type I methanotrophs primarily rely on the RuMP pathway for carbon metabolism, which is simpler and more efficient than the serine pathway utilized by type II methanotrophs. Furthermore, type I methanotrophs exhibit faster growth, with a doubling time of 2–3 h, while type II methanotrophs have a slower doubling time of 5–11 h (Kalyuzhnaya et al., 2019; Pieja et al., 2011; Puri et al., 2015). Due to these advantages, initial efforts in developing genetic tools were focused on type I methanotrophs before being extended to type II methanotrophs.

Representative model strains used in the development of genetic tools for type I methanotrophs include *Methylomicrobium buryatense*, *Methylomonas* sp. LW13, *Methylomonas* sp. DH-1, *Methylomicrobium alcaliphilum* 20Z, and *Methylococcus capsulatus* Bath.

Puri et al. (2015) were the first to report the operation of a sucrose counterselection system in type I, methanotroph, *M. buryatense*. They used this system to suppress glycogen biosynthesis by deleting two glycogen synthase genes in the *M. buryatense* chromosome. Conjugation was employed to deliver the constructed vector, using *E. coli* S17-1 as the donor strain. The experimental results showed that the sucrose counterselection system worked effectively, resulting in the mutant strain—lacking both glycogen synthase genes—producing no glycogen. Conjugation generally involves using *E. coli* (S17-1 or S18) as a donor strain to introduce plasmids containing an RK2/RP4 origin into methanotrophs (Kalyuzhnaya et al., 2015). However, this method has limitations, including a complex experimental procedure and a time-consuming process for isolating pure mutant strains. To address these challenges, Yan et al. (2016) developed an electroporation method with high transformation efficiency for the type I methanotroph *M. buryatense* 5GB1C. They optimized electroporation solvents, field strength, and recovery times, significantly improving transformation efficiency. Using this optimized electroporation method, gene knockouts or chromosomal integration of foreign gene fragments in *M. buryatense* 5GB1C were easily achieved through direct electroporation of PCR-based gene deletions or constructed plasmids. Furthermore, markerless genetic manipulations were successfully performed using FLP-FRT recombination and the sacB counterselection system. The electroporation method developed for *M. buryatense* 5GB1C has laid the groundwork for expanding genetic delivery and chromosome-editing tools for a variety of type I methanotrophs, including *Methylomonas* sp. DH-1 and *M. alcaliphilum* 20Z.

The sacB counterselection system is commonly used as a counterselection marker for removing target genes in gram-negative bacteria. However, it is not universally applicable to all gram-negative bacteria and shows low efficiency in *M. capsulatus* Bath (Ishikawa et al., 2018). To overcome this limitation, Ishikawa et al. (2018) introduced two point mutations (A306G and T252A) into the *phe*S gene, which encodes the α-subunit of phenylalanyl-tRNA synthetase in *M. capsulatus* Bath. This modification led to the creation of a new counterselection marker, *phe*S*, which enabled the successful deletion of the *xox*F gene in *M. capsulatus* Bath. Additionally, the *phe*S-based counterselection method was shown to be applicable to type II methanotrophs, such as *Methylocystis* sp. MJC1 (Kang et al., 2024b).

To enhance the genetic engineering potential of methanotrophic biocatalysts, Tapscott et al. (2019) developed a conjugation-compatible CRISPR/Cas9 genome editing system in *M. capsulatus* Bath using a series of broad-host-range expression plasmids and evaluated its efficacy. Rumah et al. (2023) were the first to demonstrate an efficient CRISPR/Cas9 system capable of generating scarless and precise gene deletions and insertions in the genomes of *M. capsulatus* Bath and *Methylocystis parvus* OBBP.

Promoters play a crucial role in the plasmid-mediated expression of foreign genes in methanotrophs. While over 30 endogenous promoters from methanotrophs have been screened, these promoters are not easily regulated, making them unsuitable for efficient genetic manipulation (Lee et al., 2021). As a result, most genetic modifications in methanotrophs depend on constitutive promoters, such as the synthetic tac promoter (P_tac_) (Nguyen et al., 2019b, 2020a, 2020b, 2020c, 2021; Nguyen & Lee, 2019, 2021). Recently, a phenol-inducible promoter has been reported for high-level expression of foreign genes in methanotroph (Jeong et al., 2023). This phenol-inducible gene expression system showed a high concentration-dependent effect in methanotrophs. Using the phenol-inducible promoter, *M. capsulatus* Bath produced up to 2 g/L of mevalonate from methane, the highest reported result for methane-derived secondary metabolites to date (Jeong et al., 2023).

## Methanol Production Using Methanotrophs as Biocatalysts

Methanol is a critical bio-based product produced from the biological conversion of methane by methanotrophs. It has been extensively studied as a precursor for commercially significant chemicals such as alcohols, formaldehyde, organic acids, and alkenes.

Methanotrophs generate methanol in the initial phase of methane oxidation. This methanol generally accumulates by inhibiting methanol dehydrogenase, the enzyme that catalyzes the conversion of methanol to formaldehyde. The activity of methanol dehydrogenase (MDH) is inhibited by calcium, EDTA, phosphate, and MgCl_2_, resulting in the buildup of methanol. This inhibition impedes later stages of methane metabolism, diminishing cellular development and resulting in an electron deficit essential for methane oxidation. Formate is introduced into the reaction medium to augment methanol synthesis, compensating for the reduced electron supply. Table 1 summerizes notable research findings from the last decade regarding methanol synthesis using methanotrophs. Hwang et al. (2015) tried to produce methanol from methane using the whole-cell form of *Methylosinus trichosporium* OB3b. They evaluated several MDH inhibitors, including EDTA and potassium phosphate, and optimized their doses (0.5 mM and 100 mM, respectively) for optimal methanol synthesis. Under optimal conditions, adding 40 mM sodium formate as a reducing agent achieved a methane-to-methanol conversion efficiency of 73.8%, producing 12.28 mM of methanol. Research has also been conducted on producing methanol by immobilizing methanotrophs. Cells of *Methylosinus sporium* covalently immobilized on chitosan demonstrated a higher efficiency in converting methane from a syngas mixture (CH_4_, CO_2_, and H_2_ ratio of 6:3:1) into methanol than free cells, yielding up to 6.12 mM of methanol (Patel et al., 2016). This catalyst exhibited markedly enhanced stability and reusability, preserving functionality for as many as six cycles under batch culture conditions. Hur et al. (2017) isolated a novel type I methanotroph, *Methylomonas* sp. DH-1, from activated sludge and optimized the batch conditions for methane-to-methanol bioconversion, including substrate concentration, biocatalyst concentration, formate concentration, and MDH inhibitors, resulting in the production of 41.86 mM methanol. *Methylomonas* sp. DH-1 showed tolerance to high methanol concentrations (up to 7% (v/v)), indicating its potential as an effective catalyst for high-yield methanol production.

A significant limitation in the biological conversion of methane using methanotrophs is the low mass transfer efficiency of methane, resulting in lower yields of the target product. Trickle-bed reactors (TBR) and continuous stirred-tank reactors (CSTR) have been employed to enhance the mass transfer efficiency of methane in methanol production (Hogendoorn et al., 2020; Sheets et al., 2017). The TBR is a cylindrical reactor filled with inert ceramic balls, where the nutrient medium circulates through the packing, forming a thin liquid layer on the packing surface. The gas can flow either co-currently or counter-currently with the liquid. In this process, the resistance to mass transfer is reduced, allowing the gas to be efficiently delivered to the biocatalysts. In the TBR, methane and oxygen mass transfer was enhanced twofold and fourfold, respectively, compared to uncompressed TBR, achieving a methanol production rate of 0.9 g/L/day (Sheets et al., 2017). MDH, a key enzyme that catalyzes methane oxidation to methanol, is categorized into *mxa*F and *xox*F types. Hogendoorn et al. (2020) investigated the accumulation of methanol by cultivating *Methylacidiphilum fumariolicum* SolV, a *Verrucomicrobia*l methanotroph that exclusively relies on *xox*F-type MDH and requires lanthanides as cofactors. The researchers used lanthanide-depleted culture media to inhibit MDH activity and promote methanol accumulation. In a CSTR reactor under lanthanide-depleted and oxygen-limited chemostat cultivation conditions, the methane conversion efficiency to methanol was 63% (mol methanol/mol methane), yielding 4.1 mM of methanol (Hogendoorn et al., 2020). The CSTR provides several advantages for optimizing methanotroph growth and producing target products. In this system, the nutrient medium is continuously supplied into the reactor so that methanotrophs are uniformly distributed without nutrient deficiency, the mass transfer is uniform, and the methane conversion efficiency is increased.

In previous studies, the accumulation of methanol using methanotrophs relied on chemical inhibitors targeting MDH, which oxidizes methanol. These inhibitors downregulated MDH activity but did not entirely stop methanol oxidation. Recently, advancements in genetic tools for methanotrophs have enabled research on methanol production by deleting MDH genes. As mentioned above, MDH in methanotrophs is classified into *mxa*F and *xox*F types. Ito et al. (2021) developed an OB3b Δ*mxa*F mutant strain deficient in *mxa*F from *M. trichosporium* OB3b and evaluated methanol accumulation with this strain. This mutant might still proliferate in environments containing cerium ions owing to the existence of an alternative MDH (*xox*F). The OB3b Δ*mxa*F mutant, when cultivated in conditions with 10 μM copper ions and 0 μM cerium ions, accumulated up to 3.36 mM of methanol. A different study group reported a *M. alcaliphilum* 20Z mutant with deletions of the *mxa*F and *xox*F genes (Le & Lee, 2023). Since *M. alcaliphilum* 20Z with deletions of both types of MDH could not grow, the deletions were attempted in a strain engineered with a glycerol utilization pathway. This strain acts as a biocatalyst, utilizing glycerol for cell growth and methane solely for the conversion to methanol. Using live cells without any chemical inhibitors of MDH or the addition of exogenous NADH, it successfully produced 11.6 mM of methanol. Additionally, resting cells supplied with 40 mM formate produced 76 mM of methanol. Most recently, a study reported the production of 132.5 mM methanol by cultivating *M. capsulatus* Bath under conditions without MDH inhibition (Priyadarsini et al., 2023).

## Production of Organic Acids by Methanotrophs

The biological conversion of methane to produce various organic acids is also being explored (Table 2). Some methanotrophs naturally produce organic acids, while others have been engineered to acquire this capability through genetic modifications.

Since 2015, progress in genomic, transcriptomic, and metabolomic investigations of methanotrophs has facilitated metabolic engineering for producing non-native organic acids. The first reported case of producing organic acids by the metabolic engineering of methanotrophs was the production of lactate. By expressing lactate dehydrogenase (LDH) in Type I methanotrophs, researchers successfully achieved methane-derived lactate production (Henard et al., 2016). Research has since focused on improving lactate yields using biogas as carbon sources (Henard et al., 2018). Nonetheless, the yield of lactate was insufficient, leading to the introduction of a synthetic biology strategy to enhance it. Through the engineering of methanotrophs using varied combinations of promoters (both constitutive and inducible) and ribosome binding sites, a strain, *M. buryatense* 5GB1 (pAMR4), was developed capable of producing L-lactate from methane at a flux 14 times greater than previously documented, achieving a lactate concentration of 0.5 g/L (Garg et al., 2018a). Lactate toxicity is a limiting factor in the production of high lactate. Lee et al (2019). conducted adaptive laboratory evolution on *Methylomonas* sp. DH-1 to generate the lactate-resistant strain JHM80. In the resistant strain, two sequence deletions were identified in the promoter region of the *wat*R gene (LysR-type transcription factor), expression of this gene was identified as partially responsible for the lactate resistance of JHM80. The *glg*A gene of the lactate-resistant strain JHM80 was removed, and a dehydrogenase gene derived from *Leuconostoc mesenteroides* subsp. mesenteroides ATCC 8293 was inserted at the relevant genomic region. JHM80 generated almost 7.5 times more d-lactate from methane compared to the *Methylomonas* sp. DH-1, reaching a concentration of 1.19 g/L.

Producing lactate from methane faces challenges such as poor carbon flux to lactate, restricted gas-liquid mass transfer, and product toxicity. Numerous studies have been undertaken to tackle these issues, and a potential strategy for improving lactic acid productivity in the future could involve blocking the conversion of pyruvate to acetyl-CoA, thereby enhancing the carbon flow toward lactate production.

Other organic acids that can be produced via the acetyl-CoA node include C-4 carboxylic acids like crotonate, and butyrate, along with succinate, muconate, 3-hydroxypropionate (3-HP), and 4-hydroxybutyrate (4HB) (Table 2). Metabolic engineering of type I methanotrophs has demonstrated successful methane-derived synthesis of C-4 carboxylic acids, including succinate and muconate. Unsaturated C-4 carboxylic acids are widely used as monomers for copolymer synthesis with broad applications in coatings, adhesives, paints, ceramics, and pesticides. Garg et al. (2018b) produced unnatural C-4 carboxylic acids, crotonate, and butyrate from methane by introducing several heterologous genes related to the reverse *β*-oxidation pathway into *M. buryatense*. They achieved production levels of 0.07 g/L for crotonate and 0.04 g/L for butyrate. Succinate derived from the TCA node serves as a platform chemical used in food, agriculture, cosmetics, and pharmaceuticals. Metabolic engineering of methanotroph, *Methylomonas* sp. DH-1 was performed to accumulate succinate (Nguyen et al., 2019b). The *sdh* gene encoding succinate dehydrogenase was deleted from the TCA cycle, glyoxylate pathway enzymes from *E. coli* MG1655 were integrated at this locus, producing the DS-GL strain. This strain achieved a maximum succinate titer of 0.195 g/L in a bioreactor. Muconate is a di-carboxylic acid that can be converted into many platform compounds. Attempts have been undertaken to produce muconate from methane by expressing heterologous genes that encode dihydroxyshikimate dehydratase, protocatechuate decarboxylase, and catechol dioxygenase in methanotrophic bacteria (Henard et al., 2019). Three methanotrophic species—*M. buryatense*, *M. capsulatus*, and *M. alcaliphilum*—were metabolically modified with three specific genes, yielding mutant strains that produced 12.4 mg/L, 0.97 mg/L, and 0.075 mg/L of muconate, respectively. In contrast, 3-hydroxypropionate and 4-hydroxybutyrate have been achieved using type II methanotrophs, *M. trichosporium* OB3b (Nguyen et al., 2020b; Nguyen and Lee, 2021).

## Methane-Derived Ectoine Production Using Methanotrophs

Ectoine is a high-value substance that can be synthesized using methanotrophs. Ectoine is a compound produced by halophilic bacteria to safeguard cells and preserve an osmotic balance in high-salinity conditions. Owing to its qualities that stabilize enzymes, nucleic acids and DNA-protein complexes, ectoine finds application in health and nutrition, although it is predominantly utilized in the cosmetics and pharmaceutical sectors (Cantera et al., 2018a).

The ectoine biosynthetic pathway initiates with acetyl-CoA and aspartate. The transformation of aspartate into L-ectoine entails a sequence of enzyme-catalyzed events mediated by three principal enzymes encoded by the *ect*ABC gene cluster: diaminobutyrate acetyltransferase (EctA), diaminobutyrate aminotransferase (EctB), and ectoine synthase (EctC) (Cantera et al., 2016; Khmelenina et al., 2015).

A research team from the Valladolid University, Spain, has done extensive ectoine production research in recent years (Table 3) (Cantera et al., 2016, 2017a, 2017b, 2018b, 2020). Cantera et al. concentrated on optimizing critical elements influencing ectoine synthesis in batch and continuous operations, examining continuous ectoine production with the established ectoine-accumulating strain *M. alcaliphilum* 20Z. Copper supplementation in the medium facilitated ectoine secretion by up to 20%, while elevated magnesium concentrations and variables such as NaCl levels augmented ectoine production (Cantera et al., 2017a, 2018b). Utilizing methane as a sole carbon source in pure cultures, ectoine was produced up to 100 mg/g biomass. Recent research has demonstrated that operating by feeding biogas during the continuous cultivation of mixed methanotrophs in a bubble column bioreactor (BCB) can achieve higher ectoine production (up to 110 mg ectoine/g biomass) without the need for magnesium or copper ion supplementation (Cantera et al., 2020).

Recently, a metabolic engineering approach for methane-derived ectoine production using methanotrophic bacteria has been reported. To enhance ectoine productivity, one strategy involved knocking out ectoine hydroxylase, which converts ectoine into its subsequent product, hydroxyectoine. Cho et al. (2022) developed *M. alcaliphilum* 20ZDP by deleting ectoine hydroxylase (*ect*D) and a transcriptional repressor of the *ect*ABC-ask operon (*ect*R) from the genome of *M. alcaliphilum* 20Z. In optimum medium conditions with 6% NaCl and 0.05 μM tungsten, the *M. alcaliphilum* 20ZDP achieved a maximum ectoine concentration of 142.32 mg/L without hydroxyectoine production. Pham et al. (2023) engineered *M. alcaliphilum* 20Z to produce ectoine using methane, glucose and xylose as co-substrate by introducing glucose and xylose utilization pathways, resulting in the mutant strain 20Z^XG^ . Cultivation of 20Z^XG^ with the three substrates upregulated the EMP and ectoine biosynthesis pathways, leading to a 1.7-fold increase in ectoine content (26.4 mg/g biomass) compared to when methane was used as the sole carbon source. Additionally, they developed the strain 20Z^XG^/Δ*ect*R by knocking out the ectoine biosynthesis repressor (*ect*R), which produced a maximum ectoine titer of 37.9 mg/g biomass in the presence of the three substrates. The effects of methane concentration and biogas on methane-derived ectoine production using methanotrophs have been reported. At a fermenter scale, utilizing a high methane concentration (O_2_/CH_4_ ratio of 1) achieved a maximum ectoine production rate of 33.8 mg/L·h, while under biogas conditions, the production rate was 19.8 mg/L·h (Choi et al., 2024).

## Methane-Derived Protein Feed Production Using Methanotrophs

Single-cell protein (SCP) means microbial protein biomass generated by various bacteria. Methanotrophs are notable as a source of SCP owing to their elevated protein content compared to fungi and yeast and their more digestible cell walls with algae (Khoshnevisan et al., 2019). Methanotroph biomass was initially sold as SCP in the late 1960s and utilized as an addition in animal feed (Bewersdorff & Dostálek, 1971; Harrison & Hamer, 1971). Currently, SCP is generated using natural gas, branded as FeedKind^®^ protein by Calysta and UniProtein^®^ by Unibio (Pieja et al., 2017). Methanotroph biomass, recognized for its elevated protein content and balanced amino acid composition, is predominantly utilized in fish feed but is also appropriate for pigs, poultry, and companion animals such as cats and dogs (Øverland et al., 2010, 2011) *M. capsulatus* and *Methylomonas* sp., the predominant methanotroph utilized for SCP production, possesses a protein concentration of up to 70% by dry cell weight (DCW) (Øverland et al., 2011; Yazdian et al., 2005). Studies have been conducted to produce SCP by recovering nutrients from industrial wastewater and co-cultivating *M. capsulatus* with other microorganisms, including *Chlorella sorokiniana*. Despite the growth of methanotrophs being limited by trace elements (e.g., copper), the protein content reached 45% of DCW (Table 4) (Rasouli et al., 2018).

Since 2019, biogas has garnered interest as an alternate methane source for SCP manufacturing. Studies indicate that biogas containing hydrogen sulfide can diminish SCP yields and amino acid concentration relative to pure methane, owing to sulfide interference in the medium (Tsapekos et al., 2019; Xu et al., 2020). Khoshnevisan et al. (2019) produced SCP using mixed methanotrophic cultures by supplying biogas and utilizing ammonium, electrochemically extracted from anaerobic digestate, as a nitrogen source. The SCP yield relative to methane reached a maximum of 0.87 g DCW/g CH_4_. Another research team supplied biogas derived from sewage sludge's anaerobic digestion (AD) to mixed methanotrophic cultures, achieving a dry weight yield of 0.66 g DCW/g CH_4_. According to reports, the biomass's protein concentration exceeded 41% (w/w) (Zha et al., 2021). The amino acid profile of methane-derived SCP differs by species; yet, it is frequently adequate to substitute traditional protein sources. Consequently, methanotroph biomass is seen as a feasible and sustainable option.

## Methane-Derived Biodegradable Plastic Production Using Methanotrophs

Biodegradable plastics are becoming recognized as potential alternatives for petroleum-based plastics, and the manufacturing of methane-derived biodegradable plastics with methanotrophs offers promising solutions for mitigating plastic waste and decreasing greenhouse gas emissions. Polyhydroxyalkanoate (PHA) is a biodegradable plastic polyester produced by methanotrophs to store compounds in excess carbon and limited nitrogen conditions. The first and most studied polyhydroxyalkanoate (PHA) is polyhydroxybutyrate (PHB), which is known for having mechanical properties similar to those of polyethylene or polypropylene. The PHB production in type II methanotrophs initiates with acetyl-CoA, subsequently including enzyme-catalyzed processes like acetyl-CoA acetyltransferase (*pha*A), acetoacetyl-CoA reductase (*pha*B), and PHA synthase (*pha*C) (Liu et al., 2020).

The production of PHB from methane has predominantly utilized species from *Methylosinus* and *Methylocystis*, with PHB accumulation yields ranging from 40% to 50% of the biomass (Lee et al., 2023; Liu et al., 2020). Sundstrom and Criddle (2015) designed a microbioreactor system and optimized calcium and copper concentrations for PHB production using pure cultures of *M. parvus* OBBP. They achieved a maximum PHB content of 49.4% and a PHB titer of 3.43 g/L (Table 5). Zhang et al. (2017) investigated the effects of various nitrogen sources on cell growth and PHB production capacity in *M. trichosporium* OB3b, which contains either sMMO or pMMO. After culturing the cells under nitrate-rich conditions, they achieved a maximum PHB content of 51% under nitrogen-limited conditions.

In recent years, much of the research on PHA production has focused on utilizing biogas. The effects of using biogas as a feedstock on the growth and polyhydroxybutyrate (PHB) synthesis of the *Methylocystis hirsuta* CSC1 were evaluated, and the highest PHB content of 45% was obtained at CH_4_:O_2_ ratios of 1:2 (Rodríguez et al., 2020a). Rodríguez et al. (2020b) also reported the inoculation of *M. hirsuta* CSC1 into a BCB, with biogas supply and gas recirculation, resulting in PHB accumulation of 14.5% of DCW. A study was conducted on the production of PHB using biogas containing hydrogen sulfide (Hyun et al., 2024). *Methylocystis* sp. MJC1 was inoculated into a bioreactor system, where biogas was supplied at a flow rate of 0.2 to 0.25 vvm (volume of gas per volume of liquid per minute). As a result of this setup, the PHB content reached 45%, and the PHB titer achieved was 2.9 g/L (Hyun et al., 2024).

Despite ongoing research into the production of PHB using methanotrophs, the productivity levels remain low. To improve the productivity of PHB derived from methane, studies have focused on high-density cell cultivation and PHB accumulation in methanotrophs. the cultivation of *Methylocystis* sp. MJC1 in a gas bioreactor, with a methane-to-air ratio of 3:7 (v/v), achieved a final cell density of 52.9 g/L and a PHB content of 53.7%, equating to 28.4 g PHB/L (Hong et al., 2024). Furthermore, using an equal ratio of methane and oxygen resulted in a PHB content of 61.7% and a concentration of 34.5 g/L, which represents the highest yield of methane-derived PHB reported to date (Hong et al., 2024).

Methanotrophs naturally accumulate PHB; however, they have limitations such as a low melting point, low thermal stability, and poor flexibility. Significant attention has been directed toward producing PHA copolymers with superior properties to PHB to improve the quality of methane-derived biodegradable plastics.

Introducing fatty acid hydrocarbon as co-substrates in the cultivation of methanotrophs leads to the successful production of PHA copolymers that exhibit increased flexibility and a lower melting point and crystallinity (Lee et al., 2023). In methanotrophs, the output of poly(3-hydroxybutyrate-co-3-hydroxyvalerate) (PHBV), which is a representative PHA copolymer, commonly involves the supply of methane and valerate as co-substrates (Lee et al., 2023; Myung et al., 2016, 2017). Adding valerate successfully induces the accumulation of PHBV copolymer incorporating 3-hydroxyvalerate (3HV). Myung et al. (2016) investigated PHBV copolymer accumulation in *M. trichosporium* OB3b to valerate concentration. By supplying methane and 100 mg/L of valerate, they achieved a PHBV copolymer content of 50% (22% of 3HV mole fraction, 0.97 g PHBV/L) (Myung et al., 2017). More recently, a relatively high yield of PHBV copolymer was achieved in a gas bioreactor by supplying *Methylocystis* sp. MJC1 with methane and valerate as co-substrates (Lee et al., 2023). The excessive supply of valerate was found to inhibit cell growth. To address this, valerate was supplied periodically at a cell density (OD_600_) above 10, producing 8.92 g PHBV/L with a 28.4% of 3HV molar fraction. The properties of the produced PHBV copolymer vary depending on the fraction of the 3HV monomer. Notably, when the 3HV fraction exceeds 25%, the material exhibits physical properties similar to polypropylene (PP) (Avella et al., 2000; Loo and Sudesh, 2007).

Some research on the metabolic engineering of methanotrophs for synthesizing PHA copolymers has been reported. Nguyen and Lee (2021) genetically modified the *M. trichosporium* OB3b to produce poly(3-hydroxybutyrate-co-4-hydroxybutyrate) [P(3HB-co-4HB)] using methane as a sole carbon source. This was accomplished by constructing a biosynthetic route for 4-hydroxybutyrate (4HB) and overexpressing CoA-transferase in *M. trichosporium* OB3b. Despite the intracellular concentration of the P(3HB-co-4HB) copolymer being only 7%, this represents the first report of PHA copolymer production using methane as a sole carbon source. Recently, an OB3b-MCRMP3S mutant strain of *M. trichosporium* OB3b was developed to enable the biosynthesis of poly(3-hydroxybutyrate-co-3-hydroxypropionate) [P(3HB-co-3HP)] copolymers (Nguyen et al., 2025). This strain produced P(3HB-co-3HP) copolymers comprising 20% of biomass when methane was used as a sole carbon source, with a 3HP molar fraction of 9%.

Currently, Mango Materials, a U.S.A company, is the only organization commercially producing PHA from methane. However, continued research and development efforts in producing methane-derived biodegradable plastics are expected to create opportunities for more companies to enter this field.

## Production of Other High-Value Products Using Methanotrophs

The research team at Kyung Hee University in Korea has been engaged in the metabolic engineering of methanotrophs to synthesize various valuable chemicals from methane while enhancing current metabolic pathways. Utilizing type I methanotrophs, *M. alcaliphilum* 20Z, and type II methanotrophs, *M. trichosporium* OB3b, as model strains, numerous study findings have been reported about the synthesis of amines and secondary metabolites alongside alcohols, organic acids, and ectoine.

Putrescine, a platform chemical monomer used in the synthesis of bioplastic nylon-4,6, has been produced from methane using an engineered strain of *M. alcaliphilum* 20ZE4-pACO developed by Nguyen and Lee (2019). The engineered strain, possessing an inactivated putrescine consumption pathway and expressing ornithine decarboxylase, achieved a maximum putrescine production of 98.08 mg/L from methane. The production of cadaverine, a crucial monomer in polyamide synthesis, has been successfully achieved using genetically modified type II methanotrophs, *M. trichosporium* OB3b (Nguyen et al., 2020c). The engineered strain, named OB3b/cad4, was designed for the production of cadaverine derived from methane. This strain successfully produced 283.63 mg/L of cadaverine using a gas bioreactor system. *M. alcaliphilum* 20Z has primarily been used as a model strain for methane-derived secondary metabolite production. Nguyen et al. (2020a, 2021) introduced biosynthetic pathways for humulene and bisabolene into *M. alcaliphilum* 20Z, resulting in the mutant strains 20Z-SQ08 and 20Z-pBs-02, respectively. Using methane as the sole carbon source, these strains successfully synthesized 0.75 mg of humulene and 12.24 mg of bisabolene. The productivity of methane-derived secondary metabolites is currently limited to the milligram scale. However, recent studies have reported improvements in precursor productivity through high-density cultivation. By optimizing fermentation conditions for an engineered strain of *M. capsulatus* Bath, a cell density of 28.2 g/L was achieved, and the mevalonate concentration derived from methane reached 1.8 g/L (Jang et al., 2023). For producing various high-value products from methane, the stability of engineered methanotroph strains and the development of appropriate fermentation technologies for each strain are expected to enhance the productivity of methane-derived high-value products in the future.

## Conclusion and Future Perspectives

Methanotrophs are promising candidates for mitigating methane emissions and producing value-added compounds because they can utilize methane—a major greenhouse gas—as a carbon source. Over the past decade, researchers have explored various methanotroph-based approaches to produce methane-derived value-added products, including alcohols, organic acids, SCP, biodegradable plastic materials, and secondary metabolites. These studies have demonstrated the feasibility of methanotrophs as cellular factories. However, challenges such as the stability of genetically modified strains, low product titers, and limited productivity constrain the industrial feasibility of methane-derived biotechnologies. Although some studies have utilized biogas as a substrate for cultivating methanotrophs and producing target products, these approaches have demonstrated lower cell growth and productivity compared to conditions that use pure methane.

Unlike *E. coli*, methanotrophs have limited genetic tools, but several type I and II methanotrophs have been genetically modified to produce value-added products. So far, efforts in the genetic engineering of methanotrophs have mainly focused on introducing 2-5 exogenous genes to convert key intermediates in methane metabolism into target products. However, there has been insufficient analysis of the transcription levels and expression strength of these introduced genes and their effects on methanotroph physiology and metabolic fluxes. A comprehensive understanding of methanotroph physiology and metabolic processes in specific environments is crucial. Multi-omics analyses are necessary to gain insights into key metabolic pathways. These insights can help identify promising target products that reflect the distinct characteristics and advantages of methanotrophs. By combining these findings with systems biology and synthetic biology techniques, we can enhance strain stability and improve the productivity of target products.

Gas fermentation technology has the potential to enhance the production of value-added products derived from methane. This requires the development of suitable bioreactor systems to enhance gas transfer efficiency, optimization of media composition and cultivation conditions to maximize productivity and the design of systems for off-gas recycling.

Currently, most studies on methane bioconversion are limited to laboratory-scale experiments that use pure methane as a carbon source. In industrial methane bioconversion, substrates like biogas or industrial by-product gases—composed of methane, carbon dioxide, carbon monoxide, alkenes, and other compounds—need to be utilized. Therefore, it is critical to screen methanotrophs capable of growing in these substrates and to evolve these strains adaptively. Such strains can serve as platforms for strain engineering to produce specific target products from methane.

Methane-derived SCP production is currently the only commercially implemented technology, though it is still in the early stages of development. Ongoing research is crucial to identifying methanotrophs with a high protein content that can grow directly on biogas and industrial by-product gases. Additionally, advancements in process engineering and design strategies are needed to facilitate large-scale SCP production that meets commercial demands and economy of scale.

For large-scale implementation of methane bioconversion using methanotrophs, a key technical challenge that must be overcome is enhancing the activity of MMO through protein engineering. Due to the low specific activity of MMO with turnover numbers of 0.16–13 s^–1^ (Lawton and Rosenzweig 2016), methanotrophs have evolved to increase MMO expression to improve the methane substrate utilization rate. It is known that MMO expression level is over 30%. However, since the availability of amino acids is limited within the cell, using a large amount of amino acids for MMO expression can reduce the amount of amino acids available for the expression of foreign genes for the production of target products. Therefore, together with improving the methane gas transfer efficiency, catalytic efficiency of MMO should be enhanced to pave the way to commercialization of methanotrophs-catalyzed methane bioconversion.

## Figures and Tables

**Table 1.** Production of methanol using methanotrophic bacteria

Strain	Substrate	Production condition	Temp (°C)	pH	Concentration (mM)	References
*M. trichosporium* OB3b	30% CH_4_	batch, 0.5 mM EDTA, 40 mM sodium formate	30	6.3	12.28	Hwang et al. (2015)
*M. sporium* (Immobilized cell on chitosan)	50% synthetic gas (CH_4_:CO_2_:H_2_=6:3:1)	batch	30	6.8	6.12	Patel et al. (2016)
*Methylomonas* sp. DH-1	40% CH_4_	batch 0.5 mM EDTA, 40 mM sodium formate	30	7.0	41.86	Hur et al. (2017)
Mixed culture (predominance of *Methylocaldum* sp. 14B)	Biogas: air = 1:2.5	trickle-bed reactor	-	-	0.9 g/L/day	Sheets et al. (2017)
*Methylacidiphilum fumariolicum* SolV	CO_2_: argon = 5:95 (v/v, 70 ml/min), CH_4_:CO_2_ = 95:5(v/v, 10 ml/min)	continuous stirred-tank reactor	55	3.0	4.1	Hogendoorn et al. (2020)
*M. trichosporium* OB3b Δ*mxa*F mutant strain	CH_4_:air 1:4 (v/v)	-	30	7.0	3.36	Ito et al. (2021)
20Z_FKDA Δ*mxa*F Δ*xox*F mutant strain	30% CH_4_	40 mM sodium formate	30	7.0	76	Le and Lee (2023)
*Methylococcus capsulatus*	50% CH_4_	without MDH inhibitors	30	6.8	132.5	Priyadarsini et al. (2023)

**Table 2.** Production of organic acid using methanotrophic bacteria

Products	Strain	Substrate	Temp (°C)	Production condition	Titer (g/L)	References
Lactate	*M. buryatense* 5GB1S pLhldh mutant strain	20% CH_4_	30	batch	0.808	Henard et al. (2016)
	*M. alcaliphilum* 20Z mutant strain	33% biogas (20% CH_4_, 13% CO_2_)	30	continuous	0.027 g/g DCW/h	Henard et al. (2018)
	pAMR4 mutant strain	21% CH_4_	30	batch	0.5	Garg et al. (2018a)
D-Lactate	*Methylomonas* sp. DH-1, JHM80 strain	20% CH_4_	30	batch	1.19	Lee et al. (2019)
Crotonate	*M. buryatense* 5GB1C strain	25% CH_4_	30	batch	0.07	Garg et al. (2018b)
Butyrate					0.04	
Succinate	*Methylomonas* sp. DH-1, DS-GL strain	30% CH_4_	30	batch	0.195	Nguyen et al. (2019b)
Muconate	*M. alcaliphilum* pMUC	20% CH_4_	30	semi-continuous	0.00075	Henard et al. (2019)
	*M. capsulatus* pMUC				0.0095	
	*M. buryatense* 5GB1 pMUC				0.012	
3-Hydroxypropionate	OB3b_MCRMP strain	30% CH_4_	30	batch	0.061	Nguyen et al. (2020b)
4-Hydroxybutyrate	OB3b_4HB-SY4 strain	30% CH_4_	30	batch	0.011	Nguyen and Lee (2021)

**Table 3.** Production of ectoine using methanotrophic bacteria

Strain	Substrate	Production condition	Temp (°C)	Titer (mg/g DCW)	References
*M. alcaliphilum* 20Z	4% CH_4_	batch, 6% NaCl, without Cu, 0.5 μM Cu	25	66.9	Cantera et al. (2016)
	20% CH_4_	batch, 6% NaCl, 50 μM Cu	30	40.7	
	4% CH_4_	continuous stirred-tank reactor, 6% NaCl, 25 μM Cu	25	37.4	Cantera et al. (2017a)
	4% CH_4_	continuous stirred-tank reactor (two sequential)	25	70.4	Cantera et al. (2017b)
	0.060 L/min CH_4_-air	bubble column reactor (continuous), pH 9.0 0.2 g/L Mg	25	94.2	Cantera et al. (2018b)
*M. alcaliphilum* 20Z, mixed haloalkaliphilic consortium	biogas (CH_4_, O_2_, CO_2_, He=31.5:55.0:13.3:0.23)	continuous, without Cu and Mg	20	0.110	Cantera et al. (2020)
*M. alcaliphilum* 20ZDP	30% CH_4_	batch, 6% NaCl, 0.05 μM W	30	142.32 mg/L	Cho et al. (2022)
*M. alcaliphilum* 20Z^XG^/ΔectR	30% CH_4_, xylose	batch, pH 9.0	30	37.9	Pham et al. (2023)

**Table 4.** Production of single cell protein using methanotrophic bacteria

Strain	Substrate	Production condition	Temp. (°C)	Intracellular protein content (%)	References
*Methylococcus capsulatus*	60% CH_4_	batch, nitrate as a N source	35	52	Pieja et al. (2017)
*M. capsulatus* (Bath), *Chlorella sorokiniana*	CH_4_: CO_2_: O_2_ = 6:3:1	batch, co-cultivation, wastewater	30	45	Rasouli et al. (2018)
Mixed methanotrophic culture (*Methylophilus* sp.)	40% CH_4_	continuous stirred-tank reactor, medium: filtered digestate	25	40.9	Tsapekos et al. (2019)
*Methylocapsa acidiphila*	60% CH_4_	batch, pH 5.7	24	58.6	Xu et al. (2020)
Mixed methanotrophic culture (*Methylomonas* and *Methylophilus* sp.)	CH_4 _: O_2_= 2:1 (v/v, biogas)	batch, medium: filtered digestate	25	41	Zha et al. (2021)

**Table 5.** Production of biodegradable plastic using methanotrophic bacteria

Strain	Substrate	Production condition	Temp. (°C)	Yields (%), g/L	References
*M. parvus* OBBP	50% CH_4_	microbioreactor system, batch, 5 μM Cu, 7.2 μM Ca, nitrogen-limited conditions	30	49.4%, 3.43 g/L	Sundstrom and Criddle (2015)
*M. trichosporium* OB3b	50% CH_4_	batch, 5 μM Cu, nitrogen-limited conditions	30	0.51	Zhang et al. (2017)
*M. hirsuta* CSC1	Synthetic biogas, O_2_:CH_4_ 2:1	batch, nitrogen-limited conditions	25	0.453	Rodríguez et al. (2020a)
*M. hirsuta* CSC1	Synthetic biogas	bubble column reactor, continuous (gas-recycling)	25	0.145	Rodríguez et al. (2020b)
*Methylocystis* sp. MJC1	Biogas:air =3:7	batch, biogas containing H_2_S	30	48%, 2.9 g/L	Hyun et al. (2024)
*Methylocystis* sp. MJC1	CH_4_:O_2 _= 1 : 1	batch, 5 μM Cu	30	61.7%, 34.5 g/L	Hong et al. (2024)
**PHBV copolymer**					
*M. trichosporium* OB3b	CH_4_:O_2_ molar ratio of 1:1.5	batch, 100 mg/L valerate	30	50% PHBV (0.97g PHBV/L), 3 HV mol fraction : 22%	Myung et al. (2016)
*M. parvus* OBBP	CH_4_:O_2_ molar ratio of 1:1.5	batch, 1.2 mM valerate	30	54% PHBV (1.0g PHBV/L), 3 HV mol fraction ; 25%	Myung et al. (2017)
*Methylocystis* sp. MJC1	CH_4_:air ratio of 3:7	batch, valerate	30	41.9% PHBV (8.92g PHBV/L), 3 HV mol fraction : 28.4%	Lee et al. (2023)
**PHA copolymer**					
*M. parvus* OBBP	CH_4_:O_2_ molar ratio of 1:1.5	batch, 7 μM Cu, co-substrates:	30	- 50% P(3HB-co-4HB), 4HB mol fraction ; 9.5%	Myung et al. (2017)
		- 4-hydroxybutyrate,		- 48% P(3HB-co-5 HV-co-3 HV), 3 HV and 5 HV mol fraction : 1.4, 3.6%	
		- 5-hydroxyvalerate,		- 48% P(3HB-co-HHxco-4HB), 4HB and 6HHx mol fraction : 1.0, 1.4 %	
*M. trichosporium* OB3b/SYOI mutant strain	40% CH_4_	batch, 5 μM Cu	30	7.01% P(3HB-co-4HB), 4HB mol fraction :3.8%	Nguyen and Lee (2021)
*M. trichosporium* MCRMP3S mutant strain	30% CH_4_	batch, 5 μM Cu	30	20% P(3HB-co-3HP), 3HP mol fraction: 9.08%	Nguyen et al. (2025)
